# Local context review by single institutional review boards: Results from a modified Delphi process

**DOI:** 10.1017/cts.2024.685

**Published:** 2024-12-18

**Authors:** Stephanie R. Morain, Juli Bollinger, Megan K. Singleton, Mia Terkowitz, Christine Weston, Jeremy Sugarman

**Affiliations:** 1 Johns Hopkins Berman Institute of Bioethics, Baltimore, MD, USA; 2 Department of Health Policy & Management, Johns Hopkins Bloomberg School of Public Health, Baltimore, MD, USA; 3 Johns Hopkins University School of Medicine, Baltimore, MD, USA; 4 Institute for Clinical and Translational Research, Johns Hopkins University, Baltimore, MD, USA

**Keywords:** Research ethics, single IRBs, local context, human subjects research, Delphi studies

## Abstract

**Introduction:**

Local context is the most common concern regarding use of a single institutional review board (sIRB). Yet what “local context” constitutes remains underspecified. Developing a shared understanding of the goals of local context review, the categories of information that should be considered, as well as the types of studies for which sIRB review may be inappropriate, are critical for ensuring that sIRB review provides adequate protections for human subjects.

**Methods:**

We conducted a three-round modified Delphi process convening individuals with expertise in the conduct and oversight of multisite research. Delphi surveys explored: (1) the goals of local context review; (2) the types of information that should be considered; and (3) study types that should be exempted from sIRB requirements.

**Results:**

Twenty-one experts participated. Experts agreed that (1) local context review should aim to both protect local participants and ensure compliance and (2) that four types of information should be considered (population/participant-level characteristics; investigator and research team characteristics; institution-level characteristics; and state and local laws). There was less consensus about whether existing processes facilitated adequate consideration of this information. Experts agreed that exemptions from sIRB requirements should be permitted but disagreed about when and in what circumstances.

**Conclusion:**

There is overlapping consensus about both the goals of local context review and the types of information that should be assessed. Future work remains, however, to develop effective processes to best realize the goals of local context review – and do so with appropriate efficiency.

## Introduction

Using a single institutional review board (sIRB) is now required for most United States (US) federally funded multisite research [1]. The most common concern regarding sIRBs relates to the need to consider the local context in which proposed research will be conducted [1]. According to the US National Institutes of Health (NIH) regulations governing the use of sIRBs, participating sites are expected to communicate “relevant information necessary for the single IRB to consider local context issues and state and regulatory requirements” [2]. Yet what, exactly, “local context issues” constitutes remains underspecified [3–5]. What are the goals of local context review? What types of information should it consider? And are there some types of research studies for which local considerations are sufficiently important or distinctive so as to make sIRB review inappropriate?

A prior scoping review explored these issues [6]. It identified five potential goals for local context review: (1) protecting the rights and welfare of local participants; (2) ensuring compliance with applicable laws and policies; (3) assessing feasibility; (4) promoting the quality of research; and (5) promoting procedural justice. It also identified four categories of information that might be considered as part of local context review: (1) population/participant-level characteristics; (2) investigator and research team characteristics; (3) institution-level characteristics; (4) state and local laws; and (5) characteristics for study exclusion from sIRB requirements.

However, the extent to which those responsible for the conduct and ethical oversight of multisite research agree on the goals and types of information that should be considered as part of local context review remains unclear. Nevertheless, a shared understanding is necessary for assessing the impact of policies mandating the use of sIRB review, including those enacted by the National Institutes of Health [2], the Revised Common Rule of 2018 [7], and a similar proposed rule by the Food and Drug Administration in 2022 [8]. Requiring review by a sIRB is predicated on the rationale that it can improve research efficiency while maintaining safeguards for research participants [9–11]. Limited evaluations suggest sIRBs may reduce the time for IRB review and study approval (although perhaps not as much as might have been anticipated) [12,13]. Less clear is their impact on participant protections, including whether sIRBs can identify and address considerations particular to the local context.

In this article, we report the results of a study employing a modified Delphi process to identify areas where there is agreement and areas where additional work is needed regarding local context review to promote efficiency and ensure protections when using a sIRB.

## Methods

**Figure 1.**
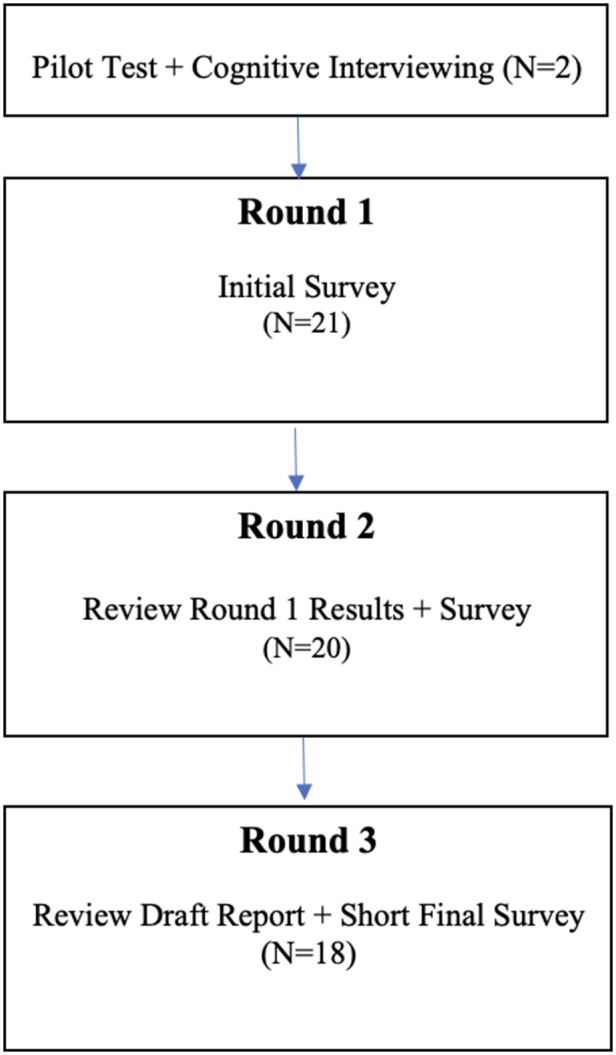
Overview of Delphi process.

We used a modified Delphi process (Fig. 1) to elicit experts’ views about local context review in a sIRB model. The Delphi process is a method of structuring group communication to synthesize expert opinion through two or more rounds of iterative surveys designed to elicit and refine experts’ views [14]. The technique has been used widely in health research [15–20], including to address issues related to research oversight [21].

Delphi panelists included individuals representing expertise in the conduct or oversight of multisite research, including current or former leaders of organizations with expertise in human subjects research (e.g., Public Responsibility in Medicine & Research, Association for the Accreditation of Human Research Protection Programs, and SMART IRB), leaders of academic human research protection programs (HRPPs), clinician investigators who have published about sIRBs, and community representatives with sIRB experience. We used purposive recruitment to obtain geographic diversity and to include relevant perspectives from academic medicine, commercial IRBs, and government. As a proxy for experience in reviewing a high volume of multisite studies, we selected academic HRPP experts from among the 30 most highly funded Clinical and Translational Science Award program hubs in fiscal year 2022, ensuring at least one representative from each of the four US census regions. We recruited participants by email and offered a $300 incentive for their estimated 4 hours of participation. The Johns Hopkins Bloomberg School of Public Health IRB determined that this study did not constitute human subjects research.

Guided by the findings of the earlier scoping review [6], we designed a survey instrument with structured response categories to elicit feedback on three issues: (1) the goals of local context review; (2) the types of information that should be considered as part of this review; and (3) whether there were any study types for which local context was so important that sIRB review would be inappropriate (and the study should therefore be exempted from sIRB requirements). Experts were also asked to provide comments explaining their answers. We pilot tested the Round 1 survey with two individuals with expertise in sIRBs. All surveys were conducted using Qualtrics. (Survey instruments provided in Supplementary Materials.)

Panelists completed Round 1 between October and November 2023. We generated descriptive statistics to determine the distribution of panelists’ answers and synthesized their free-text qualitative explanations using inductive thematic analysis.

Panelists completed the second survey in February 2024. During Round 2, we provided histograms presenting the distribution of responses to Round 1, as well as summaries of the free-text explanations. We then asked panelists to re-rate questions in light of the ratings and comments of the group and to provide qualitative comments explaining their responses. Items that received low support in Round 1 were not included in Round 2 or future evaluations (i.e., one potential goal for local context review and seven potential exemptions from sIRB requirements).

Experts completed Round 3 in April–May 2024, which involved reviewing a draft report summarizing the findings of Rounds 1 and 2 and answering a short survey. The survey solicited suggestions for improving local context review along six dimensions, with a forced choice question exploring experts’ preferences for the management of exceptions to the sIRB requirement.

## Results

Twenty-one experts participated in the Round 1 survey, 20 and 18 of whom completed the Round 2 and 3 surveys, respectively (Table 1).

**Table 1.** Panel characteristics

Characteristic	Panelists (*n* = 21)
Affiliation	
Academic	10
Commercial	3
Government	6
HRPP-focused professional organization	2
Gender	
Female	13
Male	8
Primary Role	
Investigator	5
HRPP/IRB Leader	14
Patient/Community Representative	2
Experience in the conduct or review of multisite research	
Yes	20
No	1

*Note:* HRPP, Human Research Protection Program; IRB, Institutional Review Board.

### Goals of local context review

Panelists were asked to rate how strongly they agreed with the proposed goals for local context review (Table 2). By Round 2, a substantial majority somewhat or strongly agreed that local context review should aim to *protect the rights and welfare of local participants* (18/20) and *ensure compliance with applicable laws and policies* (15/20). Views were more mixed about two other proposed goals, with 11/20 somewhat or strongly agreeing that local context review should aspire to *assess study feasibility,* and 10/20 somewhat or strongly agreeing it should *promote the quality of research*.

**Table 2.** Goals of local context review

Goal	Ratio rating as strongly or somewhat agree	
	Round 1	Round 2
Protecting the rights and welfare of (relying site) local participants	16/21	18/20
Ensuring compliance with applicable laws and policies	19/21	15/20
Assessing study feasibility	14/21	11/20
Promoting the quality of research	14/21	10/20
Promoting procedural justice*	4/21	–

Question text: To what extent do you agree or disagree that each of the following should be a goal of local context review?

* Only those items for which a majority rated as strongly/somewhat agree were included in the Round 2 survey.

When asked about the relative importance of the four goals, panelists were split as to whether *ensuring compliance with applicable laws and policies* or *protecting the rights and welfare of local (relying site) participants* was most important, with 10/20 rating the former as most important, and another 9/10 selecting the latter. Only one panelist selected *assessing study feasibility* as most important, and none selected *promoting the quality of research.*

With respect to ensuring compliance, panelists generally agreed (15/20) there needed to be a process for ensuring local site-level compliance with applicable laws and policies, and that sIRBs cannot reasonably be expected to identify and interpret relevant laws and policies across all study sites. However, they disagreed about the appropriate process for site-level review to ensure compliance with applicable laws and policies, particularly if completed by IRB staff, with some positing that this review would be better conducted through an alternative mechanism at the local institution, such as by legal counsel.

Similarly, while there was strong agreement (90%) with the general principle that protecting the rights and welfare of local participants should be a goal of local context review, a few panelists noted that rights and welfare considerations did not commonly vary across sites and expressed concern that local context review could therefore lead to unnecessary duplication of efforts.

When considering study feasibility, those who disagreed that it should be a goal of local context review argued that feasibility assessments were better managed by other entities, such as investigators or sponsors. However, others offered comments emphasizing the importance of local institutional involvement in this process, particularly in light of the high number of studies that overestimate feasibility and fail to accrue sufficient participants to address study objectives.

### Content of local context review

Most panelists strongly or somewhat agreed that all four proposed information types explored should be considered as part of local context review: population/participant-level characteristics; investigator and research team characteristics; institution-level characteristics; and state and local laws (Table 3).

**Table 3.** Content of local context review

Information type	Ratio rating as strongly or somewhat agree
Population/participant-level characteristics	18/21
Investigator and research team characteristics	16/21
Institution-level characteristics	18/21
State and local laws	19/21

Question text: To what extent do you agree or disagree that each of the following should be considered as part of local context review?

However, there was less agreement as to whether current processes for local context review facilitated adequate consideration of these information types (Table 4). While at least 70% agreed or strongly agreed that local context review processes facilitated adequate consideration of state and local laws (16/20), and institution characteristics (14/20), fewer expressed confidence that these processes did so for investigator and research team characteristics (12/20), or population/participant-level characteristics (9/20).

**Table 4.** Appropriateness of existing processes to assess relevant local characteristics

Information type	Ratio rating as strongly or somewhat agree
Population/participant-level characteristics	9/20
Investigator and research team characteristics	12/20
Institution-level characteristics	14/20
State and local laws	16/20

Question text: To what extent do you agree or disagree that current processes for local context review facilitate appropriate consideration for each of the following?

At least four specific concerns related to processes for assessing population/participation-level characteristics were identified: (1) uncertainty about which specific characteristics should be considered; (2) a lack of standardized tools or processes for identifying relevant information; (3) uncertainty about who should review information (e.g., IRB staff versus IRB members); and (4) that staffing at many IRBs was insufficient to facilitate adequate review.

Additional concerns related to assessing the other three information types included those pertaining to: researchers with a history of compliance or disciplinary issues; local site resources or local standards of care and, whether IRB staff were best positioned to assess and communicate these considerations; and whether existing processes facilitated appropriate interpretation of whether and how identified laws might apply to a specific study.

### Potential exceptions to sIRB requirements

Overall, there was little consensus about the appropriateness of exemptions to sIRB requirements (Table 5). Of the thirteen study types initially explored, by Round 2, only one received support from a slight majority of panelists as being appropriate for an exemption: non-clinical studies in which sites are not conducting the same research activities (12/20). Sizable minorities supported exemptions for three additional study types, including studies involving: unique ethnic or religious groups (8/20); studies that are not clinical trials (7/20); and studies operating under the Food and Drug Administration’s Exception from the Requirement to Obtain Informed Consent (7/20).

**Table 5.** Potential study-specific exceptions from the sIRB requirement

Study type	Ratio rating as strongly/somewhat agree	
	Round 1	Round 2*
Studies involving gene therapy or stem cells	2/21	–
First-in-human, Phase I/II studies	3/21	–
Surgical studies	4/21	–
Research involving:		
…unique ethnic or religious groups	6/21	8/20
…individuals with stigmatized conditions	1/21	–
… prisoners	5/21	–
… marginalized groups	5/21	–
… a small number of sites (e.g., <5)	11/21	5/20
… minimal risk studies	8/21	3/20
…studies that are not clinical trials	8/21	7/20
…non-clinical studies in which sites are not conducting the same research activities	13/21	12/20
… researchers/teams with a history of compliance issues	2/21	–
…studies operating under EFIC (Exception from Informed Consent Requirements for Emergency Research)	10/21	7/20

Question text: To what extent do you agree or disagree that each of the following should be granted an exception from the single IRB requirement?

* Only those items for which a majority rated as strongly/somewhat agree or unsure were included in the Round 2 survey.

Notably, for the six study types explored in Round 2, at least one panelist “strongly agreed” that the study merited an exemption, and at least one “strongly disagreed,” suggesting continued dissensus regarding the appropriateness of study-specific exemptions from sIRB requirements.

These disagreements about the details of exemptions notwithstanding, there was consensus that exemptions from the sIRB requirements should be permitted. When asked in Round 3 about how exemption determinations should be made, a slight majority (11/18) preferred a case-by-case basis rather than categorical exemptions for all studies of a certain type (e.g., those involving stem cells or those involving no more than minimal risk).

## Discussion

This study provides important and novel insights into the views of experts in the conduct or oversight of multisite research with human subjects about local context review for sIRBs. Three themes merit particular consideration: (1) lack of agreement of the goals of local context review; (2) whether current processes fit the goals of local context review; and (3) when exceptions should be permitted.

First, consistent with the findings of the earlier scoping review [6], experts do not fully agree about the goals of local context review or their relative prioritization. Our data found strong support for the view that local context review should aim to protect the rights and welfare of local participants and to ensure compliance with applicable laws and policies. However, panelists held mixed views about whether local context review should aim to ensure feasibility or promote the quality of research. Furthermore, panelists were split as to the relative priority of protecting rights and welfare versus ensuring compliance. This divergence may impair ongoing efforts to design appropriate processes for local context review, as well as assessments of their effectiveness.

Second, questions remain about the fit – or lack thereof – between goals of local context review and existing processes for their fulfillment. Our finding that the majority of panelists viewed existing processes for assessing state and local laws as adequate provides some confidence that these processes can be supportive of the goal of ensuring compliance. Nevertheless, some compliance-related concerns remain, including how best to ensure that sIRBs are not only aware of relevant laws but also have guidance about how to appropriately interpret them in specific research contexts. Moreover, the fact that a strong majority of panelists viewed protecting participants’ rights and welfare as an important goal of local context review, yet a substantial portion of panelists found current processes for assessing participant- and investigator-level characteristics as lacking suggests the need to refine these processes to ensure this goal is realized. Potential next steps to support this effort include developing greater standardization of the specific types of information that are relevant for making these assessments (e.g., which particular participant- and/or investigator-level characteristics should be considered), as well as the processes by which to communicate and assess that information. As part of this effort, more attention is needed to the question of who should be engaged in various components of the review process (both at the relying organization and reviewing IRB), including which components require expertise beyond that of administrative IRB staff members, and, relatedly, which types of considerations might benefit from broader expertise and deliberation, such as that which might occur via a convened review of the full IRB.

Third, while we found broad agreement that some studies should be exempted from the requirement to use a sIRB, there remains far less clarity as to when and in what circumstances such exemptions should be permitted. Yet requiring the use of a sIRB is not without possible drawbacks, including that doing so may delay, rather than accelerate, the time to approval [4], or might undermine important protections for human subjects. Prior calls have been made for the NIH to convene an expert panel to develop criteria to inform assessments of when exemptions might be appropriate [22]. The divergence in views among our panelists about the appropriateness of exemptions underscores the potential value of this or similar opportunities for deliberation and guidance development.

Despite the importance of our findings, several limitations merit consideration. First, like all studies involving a Delphi process, our findings are dependent upon the composition of the expert panel. We deliberately assembled an expert panel with a broad range of perspectives: individuals representing commercial, government, and non-academic IRBs, as well as those with expertise beyond the IRB/HRPP, including investigators and patient or community representatives. While our experts had extensive experience in the conduct and/or oversight of multisite research, including under a sIRB, the results of our process may not be representative of all relevant perspectives. Second, while our survey was informed by a scoping review, there may be potential characteristics within the categories that we did not fully explore. Third, space limitations within the Delphi surveys precluded our ability to examine some characteristics at the level of granularity needed to inform future practice (e.g., identifying the specific types of information related to patient- or population-level characteristics that should be considered).

## Conclusion

How sIRBs should consider local context remains a leading concern about sIRB review for multisite studies. This study is the first to characterize areas of agreement and disagreement among multidisciplinary experts regarding the goals and content of local context review and about potential exemptions to federal sIRB requirements. Our findings suggest shared agreement that local context review should aim to both protect the rights and welfare of local participants and ensure compliance. They also suggest that information related to characteristics of patients, populations, investigators, and institutions are relevant to assessing local context, as are state and local laws. However, future work remains to develop effective processes to best realize the goals of local context review and to do so with appropriate efficiency.

## Supporting information

Morain et al. supplementary material Morain et al. supplementary material

## Supplementary material

The supplementary material for this article can be found at https://doi.org/10.1017/cts.2024.685
