# Supplementary material for: Local context review by single institutional review boards: Results from a modified Delphi process
Source: J Clin Transl Sci. 2024 Dec 18;9(1):e2. doi: 10.1017/cts.2024.685 (PMC11736292; doi:10.1017/cts.2024.685)
Supplement: Morain et al. supplementary material [file S205986612400685Xsup001.docx]

Delphi Round 1

Start of Block: Default Question Block

**INTRODUCTION**

Thank you for agreeing to participate in our Delphi study of local context review by single IRBs. The input that you provide through this study is very important and will be used to develop guidance for ethical and efficient single IRB review. 

As a reminder, this Delphi study will include two survey “rounds” and a review of draft guidance documents. This initial survey, Survey 1, is divided into three sections, focusing on the following topics:

Section 1: Goals of local context (4 questions) 
Section 2: Domains of local context review (5 questions) 
Section 3: Permissible exceptions from sIRB Requirements (13 questions) 

At the end of each section we ask you an additional question about whether there is anything we missed that you would like to add. 

Within each section, you will be asked to provide a rating on a scale from 'Strongly disagree' to 'Strongly agree'. For Section 1, you may be prompted to provide feedback to share your reasoning for your ratings. For Sections 2 and 3, you will be prompted to provide feedback for each of your ratings.

For a Delphi survey, the reason that you provide for your answer is as important as the rating itself. Therefore, the survey will prompt you to share your reasoning in the text boxes provided.

As a reminder, your participation in this study is not anonymous, however your responses will be kept confidential. In subsequent survey rounds, we may share excerpts from survey responses, but we will not attribute specific responses to any individual. The final report resulting from this study will only provide aggregate results and will be de-identified.

| Page Break |  |
| --- | --- |

**INSTRUCTIONS**
 
Please complete this survey on a computer (as opposed to your phone) due to the length and complexity of the survey. 

You are permitted to page back and forth in the survey using the arrows at the bottom. 

According to our pilot study, it should take you approximately 45 minutes to an hour to complete this study. 

We recommend that you complete this survey in one sitting. However, if you need to leave the survey and return to it at another time, you may do so by leaving the survey and re-clicking on the link in your email when you are ready to return. All of the work that you have done up to that point will have been saved. 

Your careful attention and commitment to a thoughtful response is greatly appreciated. As an expression of our appreciation for your time and input, you will be reimbursed $100 for each survey.

If you have any questions about this survey, please reach out to Stephanie Morain at smorain1@jhu.edu.

| Page Break |  |
| --- | --- |

**PART 1: Goals of Local Context** 
 
What are the goals of local context review?

Various processes have been developed to facilitate “local context review” as part of single IRB review, or the process by which individuals or committees at each participating site or institution evaluate the ethical and feasibility considerations relevant to the particular local context at their respective site. However, there is not yet shared agreement as to the purpose or goals of local context review, which complicates the ability to assess these approaches.

For the following 5 questions, please rate the extent to which you agree or disagree that each of the following should be a goal of local context review. After each rating you will be invited to share the reason for your rating.

| Page Break |  |
| --- | --- |

1. To what extent do you agree or disagree that **protecting the rights and welfare of (relying site) local participants** should be a goal of local context review?

- Strongly disagree
- Somewhat disagree
- Unsure
- Somewhat agree
- Strongly agree

Display This Question:

If 1. To what extent do you agree or disagree that protecting the rights and welfare of (relying sit... = Somewhat disagree

Or 1. To what extent do you agree or disagree that protecting the rights and welfare of (relying sit... = Strongly disagree

Or 1. To what extent do you agree or disagree that protecting the rights and welfare of (relying sit... = Unsure

Please provide comments to explain your reasoning for your response.

___________________________________________________________

___________________________________________________________

___________________________________________________________

___________________________________________________________

___________________________________________________________

| Page Break |  |
| --- | --- |

2. To what extent do you agree or disagree that **ensuring compliance with applicable laws and policies** should be a goal of local context review?

- Strongly disagree
- Somewhat disagree
- Unsure
- Somewhat agree
- Strongly agree

Display This Question:

If 2. To what extent do you agree or disagree that ensuring compliance with applicable laws and poli... = Strongly disagree

Or 2. To what extent do you agree or disagree that ensuring compliance with applicable laws and poli... = Somewhat disagree

Or 2. To what extent do you agree or disagree that ensuring compliance with applicable laws and poli... = Unsure

Please provide comments to explain your reasoning for your response.

___________________________________________________________

___________________________________________________________

___________________________________________________________

___________________________________________________________

___________________________________________________________

| Page Break |  |
| --- | --- |

3. To what extent do you agree or disagree that **assessing study feasibility** (e.g., resources, staff, likelihood of meeting study accrual targets) should be a goal of local context review?

- Strongly disagree
- Somewhat disagree
- Unsure
- Somewhat agree
- Strongly agree

Display This Question:

If 3. To what extent do you agree or disagree that assessing study feasibility (e.g., resources, sta... = Strongly disagree

Or 3. To what extent do you agree or disagree that assessing study feasibility (e.g., resources, sta... = Somewhat disagree

Or 3. To what extent do you agree or disagree that assessing study feasibility (e.g., resources, sta... = Unsure

Please provide comments to explain your reasoning for your response.

___________________________________________________________

___________________________________________________________

___________________________________________________________

___________________________________________________________

___________________________________________________________

| Page Break |  |
| --- | --- |

4. To what extent do you agree or disagree that **promoting the quality of research** *(for example, by tailoring research measures to be sensitive to cultural needs while maintaining scientific integrity)* should be a goal of local context review?

- Strongly disagree
- Somewhat disagree
- Unsure
- Somewhat agree
- Strongly agree

Please provide comments to explain your reasoning for your response.

___________________________________________________________

___________________________________________________________

___________________________________________________________

___________________________________________________________

___________________________________________________________

| Page Break |  |
| --- | --- |

5. To what extent do you agree or disagree that **promoting procedural justice** (*via improved accountability or accessibility of local review, as compared to centralized review*) should be a goal of local context review?

- Strongly disagree
- Somewhat disagree
- Unsure
- Somewhat agree
- Strongly agree

Please provide comments to explain your reasoning for your response.

___________________________________________________________

___________________________________________________________

___________________________________________________________

___________________________________________________________

___________________________________________________________

| Page Break |  |
| --- | --- |

6. Are there additional goals, beyond those which we have asked you about here, that you think should be considered as goals of local context review?

As a reminder, the goals that were listed in this section were:

• Protecting the rights and welfare of local participants;

• Ensuring compliance with applicable laws and policies;

• Assessing study feasibility;

• Promoting the quality of research;

• Promoting procedural justice.

If there are other goals that you believe should be considered, please describe them and why they are relevant for local context review. If you believe there are no additional goals, feel free to let us know that as well.

___________________________________________________________

___________________________________________________________

___________________________________________________________

___________________________________________________________

___________________________________________________________

| Page Break |  |
| --- | --- |

PART 2: Domains of Local Context

What types of information should be considered as part of local context review?

While several types of information have been proposed as relevant for local context review, there is not yet agreement about what information should be considered by sIRBs.

For the next 4 questions, please rate the extent to which you agree or disagree that each of the following should be considered as part of local context review.

After each rating you will be invited to share the reason for your rating.

| Page Break |  |
| --- | --- |

1. To what extent do you agree or disagree that **population/participant-level characteristics (e.g., demographics, culture & religion, language & literacy, etc.)** should be considered as part of local context review?

- Strongly disagree
- Somewhat disagree
- Unsure
- Somewhat agree
- Strongly agree

Please provide comments to explain your reasoning for your response.

___________________________________________________________

___________________________________________________________

___________________________________________________________

___________________________________________________________

___________________________________________________________

| Page Break |  |
| --- | --- |

2. To what extent do you agree or disagree that **investigator and research team characteristics (e.g., competency and training, credentialing, conflicts of interest, history of compliance issues or disciplinary actions, etc.)** should be considered as part of local context review?

- Strongly disagree
- Somewhat disagree
- Unsure
- Somewhat agree
- Strongly agree

Please provide comments to explain your reasoning for your response.

___________________________________________________________

___________________________________________________________

___________________________________________________________

___________________________________________________________

___________________________________________________________

| Page Break |  |
| --- | --- |

3. To what extent do you agree or disagree that **institution-level characteristics (e.g., local standards of care, institutional policies, site-level resources and capabilities, etc.)** should be considered as part of local context review?

- Strongly disagree
- Somewhat disagree
- Unsure
- Somewhat agree
- Strongly agree

Please provide comments to explain your reasoning for your response.

___________________________________________________________

___________________________________________________________

___________________________________________________________

___________________________________________________________

___________________________________________________________

| Page Break |  |
| --- | --- |

4. To what extent do you agree or disagree that **state and local laws (e.g., laws pertaining to age and decision-making, confidentiality, mandatory reporting, etc.)** should be considered as part of local context review?

- Strongly disagree
- Somewhat disagree
- Unsure
- Somewhat agree
- Strongly agree

Please provide comments to explain your reasoning for your response.

___________________________________________________________

___________________________________________________________

___________________________________________________________

___________________________________________________________

___________________________________________________________

| Page Break |  |
| --- | --- |

5. Are there other types of information that should be included as part of local context review?

As a reminder, the information types listed in this section were:

 • Population/participant-level characteristics;

 • Investigator and research team characteristics;

 • Institution-level characteristics;

 • State and local laws.

If there are other types of information, please describe the type of information and why it is important below. If you have no additional types of information to add, feel free to let us know that as well.

___________________________________________________________

___________________________________________________________

___________________________________________________________

___________________________________________________________

___________________________________________________________

| Page Break |  |
| --- | --- |

**PART 3: Permissible Exceptions from sIRB Requirements**
 
When, if at all, should studies be exempted from single IRB requirements?

Leading professional organizations have called for the development of criteria by which to assess if and when studies might be granted an exception from the requirements for single IRB review.

For the next 13 questions, please rate the extent to which you agree or disagree that the type of study described should NOT be required to use a single IRB.

After each rating you will be invited to share the reason for your rating.

| Page Break |  |
| --- | --- |

1. To what extent do you agree or disagree that studies involving **gene therapy or stem cells** should be granted an exception from the single IRB requirement?

- Strongly disagree
- Somewhat disagree
- Unsure
- Somewhat agree
- Strongly agree

Please provide comments to explain your reasoning for your response.

___________________________________________________________

___________________________________________________________

___________________________________________________________

___________________________________________________________

___________________________________________________________

| Page Break |  |
| --- | --- |

2. To what extent do you agree or disagree that **first-in-human, Phase I/II studies** should be granted an exception from the single IRB requirement?

- Strongly disagree
- Somewhat disagree
- Unsure
- Somewhat agree
- Strongly agree

Please provide comments to explain your reasoning for your response.

___________________________________________________________

___________________________________________________________

___________________________________________________________

___________________________________________________________

___________________________________________________________

| Page Break |  |
| --- | --- |

3. To what extent do you agree or disagree that **surgical studies** should be granted an exception from the single IRB requirement?

- Strongly disagree
- Somewhat disagree
- Unsure
- Somewhat agree
- Strongly agree

Please provide comments to explain your reasoning for your response.

___________________________________________________________

___________________________________________________________

___________________________________________________________

___________________________________________________________

___________________________________________________________

| Page Break |  |
| --- | --- |

4. To what extent do you agree or disagree that research involving **unique ethnic or religious groups** should be granted an exception from the single IRB requirement?

- Strongly disagree
- Somewhat disagree
- Unsure
- Somewhat agree
- Strongly agree

Please provide comments to explain your reasoning for your response.

___________________________________________________________

___________________________________________________________

___________________________________________________________

___________________________________________________________

___________________________________________________________

| Page Break |  |
| --- | --- |

5. To what extent do you agree or disagree that research involving **individuals with stigmatized conditions** *(e.g., HIV, substance use, etc.)* should be granted an exception from the single IRB requirement?

- Strongly disagree
- Somewhat disagree
- Unsure
- Somewhat agree
- Strongly agree

Please provide comments to explain your reasoning for your response.

___________________________________________________________

___________________________________________________________

___________________________________________________________

___________________________________________________________

___________________________________________________________

| Page Break |  |
| --- | --- |

6. To what extent do you agree or disagree that research involving **prisoners** should be granted an exception from the single IRB requirement?

- Strongly disagree
- Somewhat disagree
- Unsure
- Somewhat agree
- Strongly agree

Please provide comments to explain your reasoning for your response.

___________________________________________________________

___________________________________________________________

___________________________________________________________

___________________________________________________________

___________________________________________________________

| Page Break |  |
| --- | --- |

7. To what extent do you agree or disagree that research involving **marginalized groups, including those with historically contentious relationships with research(ers)** should be granted an exception from the single IRB requirement?

- Strongly disagree
- Somewhat disagree
- Unsure
- Somewhat agree
- Strongly agree

Please provide comments to explain your reasoning for your response.

___________________________________________________________

___________________________________________________________

___________________________________________________________

___________________________________________________________

___________________________________________________________

| Page Break |  |
| --- | --- |

8. To what extent do you agree or disagree that research involving **a small number of sites (e.g., fewer than 5)** should be granted an exception from the single IRB requirement?

- Strongly disagree
- Somewhat disagree
- Unsure
- Somewhat agree
- Strongly agree

Please provide comments to explain your reasoning for your response.

___________________________________________________________

___________________________________________________________

___________________________________________________________

___________________________________________________________

___________________________________________________________

| Page Break |  |
| --- | --- |

9. To what extent do you agree or disagree that research involving **minimal risk studies** should be granted an exception from the single IRB requirement?

- Strongly disagree
- Somewhat disagree
- Unsure
- Somewhat agree
- Strongly agree

Please provide comments to explain your reasoning for your response.

___________________________________________________________

___________________________________________________________

___________________________________________________________

___________________________________________________________

___________________________________________________________

| Page Break |  |
| --- | --- |

10. To what extent do you agree or disagree that research involving **research studies that are not clinical trials, including epidemiological or social science research,** should be granted an exception from the single IRB requirement?

- Strongly disagree
- Somewhat disagree
- Unsure
- Somewhat agree
- Strongly agree

Please provide comments to explain your reasoning for your response.

___________________________________________________________

___________________________________________________________

___________________________________________________________

___________________________________________________________

___________________________________________________________

| Page Break |  |
| --- | --- |

11. To what extent do you agree or disagree that research involving **non-clinical studies in which the sites are not conducting the same research activities** should be granted an exception from the single IRB requirement?

- Strongly disagree
- Somewhat disagree
- Unsure
- Somewhat agree
- Strongly agree

Please provide comments to explain your reasoning for your response.

___________________________________________________________

___________________________________________________________

___________________________________________________________

___________________________________________________________

___________________________________________________________

| Page Break |  |
| --- | --- |

12. To what extent do you agree or disagree that research involving **researchers/teams with a history of compliance issues** should be granted an exception from the single IRB requirement?

- Strongly disagree
- Somewhat disagree
- Unsure
- Somewhat agree
- Strongly agree

Please provide comments to explain your reasoning for your response.

___________________________________________________________

___________________________________________________________

___________________________________________________________

___________________________________________________________

___________________________________________________________

| Page Break |  |
| --- | --- |

13. To what extent do you agree or disagree that research involving **studies operating under EFIC (exception from informed consent for emergency research)** should be granted an exception from the single IRB requirement?

- Strongly disagree
- Somewhat disagree
- Unsure
- Somewhat agree
- Strongly agree

Please provide comments to explain your reasoning for your response.

___________________________________________________________

___________________________________________________________

___________________________________________________________

___________________________________________________________

___________________________________________________________

| Page Break |  |
| --- | --- |

14. Are there additional characteristics or study types, beyond those listed here, that should qualify a study for an exception from the requirements to use a sIRB?

As a reminder, the characteristics or study types listed in this section were:

- gene therapy or stem cells;
- first-in-human, Phase I/II studies;
- surgical studies;
- unique ethnic or religious groups;
- individuals with stigmatized conditions;
- prisoners;
- marginalized groups, including those with historically contentious relationships with IRBs or with research;
- a small # of sites; minimal risk studies, including studies initially eligible for expedited review;
- research studies that are not clinical trials, including epidemiological, public health, or social science research;
- non-clinical studies in which the sites are not conducting the same research activities;
- researchers/teams with a history of compliance issues;
- studies operating under EFIC.

  If there other study types for which you believe sIRB review would NOT be appropriate, please describe the characteristic and the rationale below. If you have no additional study types to add, feel free to let us know that as well.

___________________________________________________________

___________________________________________________________

___________________________________________________________

___________________________________________________________

___________________________________________________________

| Page Break |  |
| --- | --- |

Are there other issues related to local context review, beyond those that we have asked you about during this survey, that you think are important to consider? If so, please share your thoughts below.

___________________________________________________________

___________________________________________________________

___________________________________________________________

___________________________________________________________

___________________________________________________________

| Page Break |  |
| --- | --- |

**Background Characteristics**

**Affiliation** (please specify in the open text box)

- Academic (state institution) __________________________________________________
- Academic (private institution) __________________________________________________
- Commercial IRB __________________________________________________
- Government IRB __________________________________________________
- Other __________________________________________________

| Page Break |  |
| --- | --- |

**Role:** Which of the following best describes your role? (select ALL that apply)

- Institutional Review Board (IRB) staff
- Institutional Review Board (IRB) member or chairperson
- Human Research Protection Program (HRPP) staff or leader
- Investigator
- Institutional/health system leader
- Patient/community advocate
- Other __________________________________________________

| Page Break |  |
| --- | --- |

Have you been involved in the conduct or review of multi-site studies using a single IRB review?

- Yes
- No
- Unsure

Display This Question:

If Have you been involved in the conduct or review of multi-site studies using a single IRB review? = Yes

If yes, experience as:

- Reviewing site (the institution or organization designated as being responsible for overseeing the ethical review and approval of a research study involving multiple sites)
- Relying site (the individual institutions or organizations that collaborate in a multi-site research activity)
- Both
- Other (please explain) __________________________________________________

| Page Break |  |
| --- | --- |

Have you received training or other guidance specific to the new sIRB requirements? (For example, attended a webinar or in-person training, read a guidance document, etc.)

- Yes
- No

Display This Question:

If Have you received training or other guidance specific to the new sIRB requirements? (For example,... = Yes

What training type(s) did you receive? Please select all that apply.

- Webinar
- E-learning or online training
- Guidance or workshop session (in-person or virtual)
- Guidance Document
- Other __________________________________________________

| Page Break |  |
| --- | --- |

Do you consider yourself to be Hispanic or Latino?

- No
- Yes
- Prefer not to answer

| Page Break |  |
| --- | --- |

What race or races do you consider yourself to be? Please select one or more of these categories.

- American Indian or Alaska Native
- Asian
- Black or African American
- Native Hawaiian or Pacific Islander
- White or Caucasian
- Prefer not to answer

| Page Break |  |
| --- | --- |

Gender: How do you identify?

- Male
- Female
- Trans gender female or Trans women
- Trans gender male or Trans male
- Non-binary, Genderqueer, or Genderfluid
- Something else
- Prefer not to say

End of Block: Default Question Block

Delphi Round 2

Start of Block: Default Question Block

**INTRODUCTION**

Thank you for completing the first survey in this Delphi study of local context review by single IRBs. Your ratings and comments are invaluable to this process.  

In this second round, you will be asked to do three things:

1. REVIEW the ratings and comments from selected items from the Round 1 survey.
2. RE-RATE each question, taking into consideration the responses and comments of your peers.
3. MAKE COMMENTS on any question.

| Page Break |  |
| --- | --- |

**INSTRUCTIONS**
 
Please complete this survey on a computer (as opposed to your phone) due to the length and complexity of the survey.

You are permitted to page back and forth in the survey using the arrows at the bottom.

According to our pilot study, it should take you approximately 45 minutes to an hour to complete this study.

We recommend that you complete this survey in one sitting. However, if you need to leave the survey and return to it at another time, you may do so by leaving the survey and re-clicking on the link in your email when you are ready to return. All of the work that you have done up to that point will have been saved.

Your careful attention and commitment to a thoughtful response is greatly appreciated. As an expression of our appreciation for your time and input, you will be reimbursed $100 for this survey.

If you have any questions about this survey, please reach out to Stephanie Morain at smorain1@jhu.edu.  

| Page Break |  |
| --- | --- |

**PART 1: Goals of Local Context**

In Round 1, you were asked to consider the goals of local context review. You were also asked to provide comments to explain your ratings.

In this round, we ask you to review the distribution of respondent answers for selected items, along with a summary of reasons offered by your peers and/or from the literature supporting the different views.

After considering the responses and comments for your peers from Round 1, we will ask you to re-rate each question.

| Page Break |  |
| --- | --- |

Question: To what extent do you agree or disagree that **protecting the rights and welfare of (relying site) local participants** should be a goal of local context review?

Round 1 Responses:


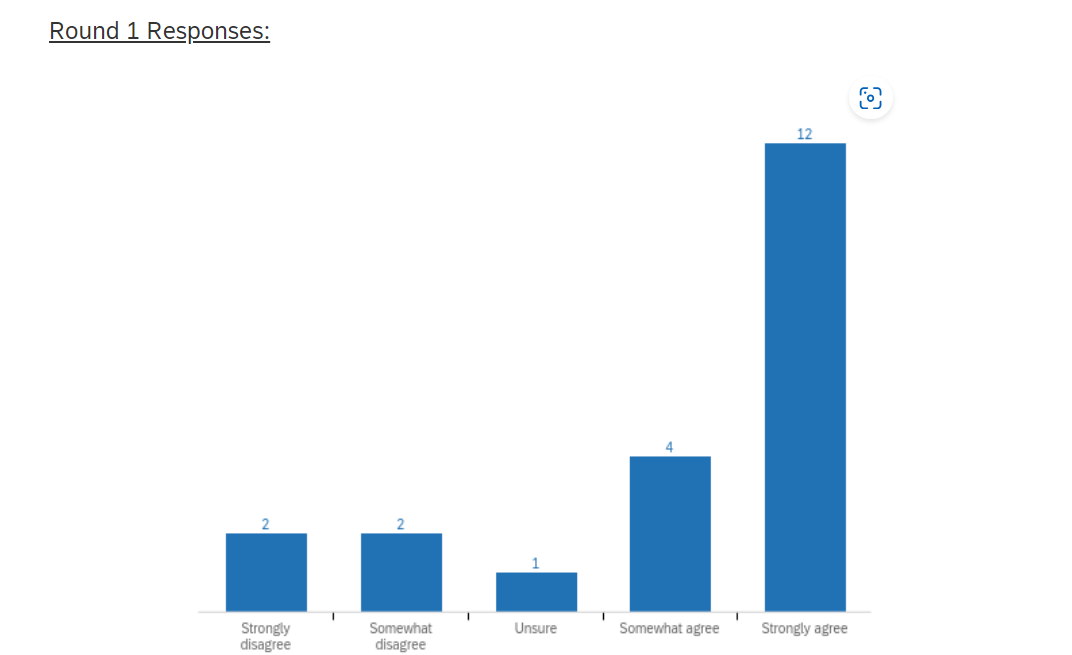


| **Qualitative Comments:** | |
| --- | --- |
| **Reasons for why it should NOT be a goal of LCR** | **Reasons for why it SHOULD be a goal of LCR** |
| - LCR is duplicative of the work of the IRB of record (reviewing IRB) - The rights and welfare of participants does not vary by site or does so only rarely | - Differences in local population characteristics and/or local site resources may influence assessment of risks and benefits - Local knowledge is important for ensuring that advertisements, recruitment letters, and incentives are culturally appropriate so as to promote autonomy and minimize undue influence |

| Page Break |  |
| --- | --- |

1.1 Now that you’ve reviewed this information, please re-rate your response.
 
To what extent do you agree or disagree that **protecting the rights and welfare of (relying site) local participants** should be a goal of local context review?

- Strongly disagree
- Disagree
- Unsure
- Agree
- Strongly agree

Please provide comments to explain your reasoning for your response (optional).

________________________________________________________________

________________________________________________________________

________________________________________________________________

________________________________________________________________

________________________________________________________________

| Page Break |  |
| --- | --- |

Question: To what extent do you agree or disagree that **ensuring compliance with applicable laws and policies** should be a goal of local context review?

Round 1 Responses:


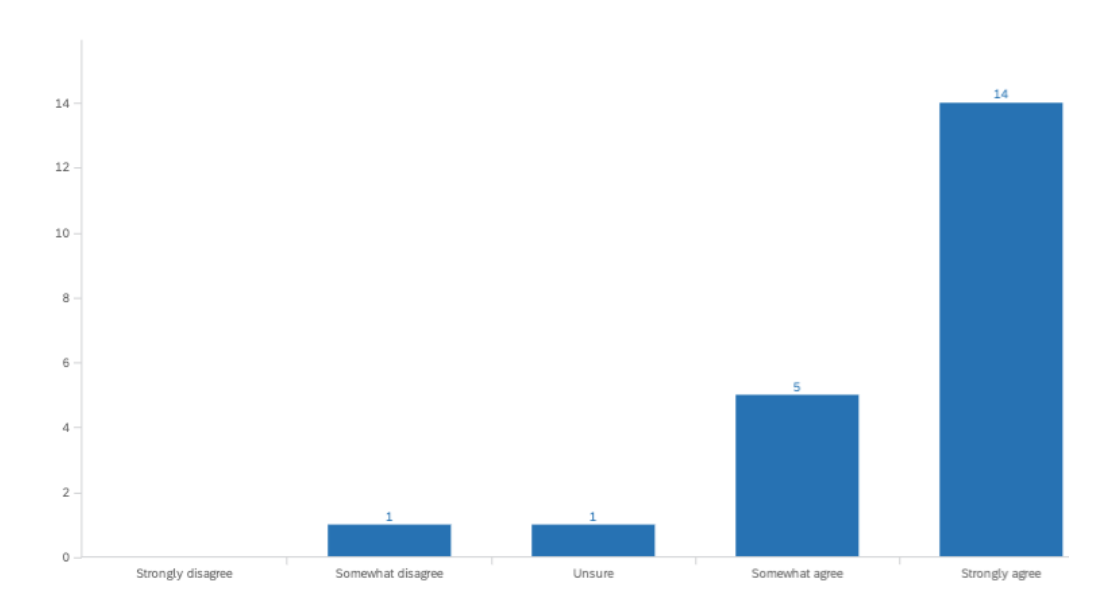


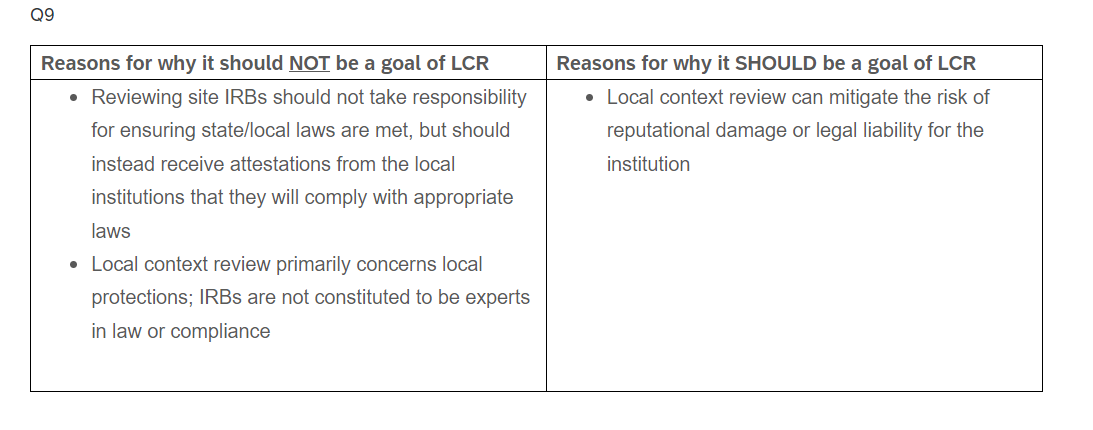


1.2 Now that you’ve reviewed this information, please re-rate your response.

To what extent do you agree or disagree that **ensuring compliance with applicable laws and policies** should be a goal of local context review?

- Strongly disagree
- Disagree
- Unsure
- Agree
- Strongly agree

Please provide comments to explain your reasoning for your response (optional).

________________________________________________________________

________________________________________________________________

________________________________________________________________

________________________________________________________________

________________________________________________________________

| Page Break |  |
| --- | --- |

Question: To what extent do you agree or disagree that **assessing study feasibility** (e.g., resources, staff, likelihood of meeting study accrual targets) should be a goal of local context review?

Round 1 Responses:


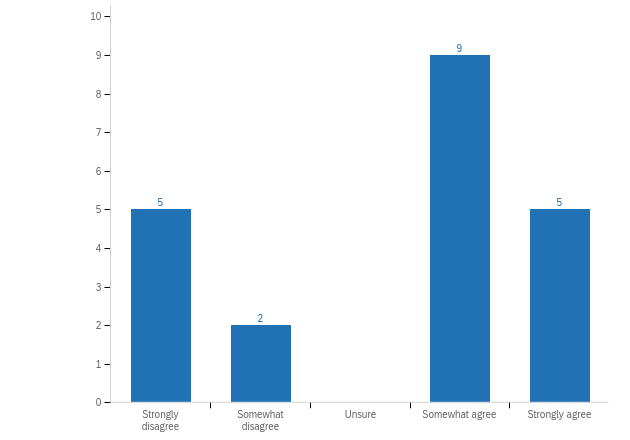


| **Qualitative Comments:** | |
| --- | --- |
| **Reasons for why it should NOT be a goal of LCR** | **Reasons for why it SHOULD be a goal of LCR** |
| - LCR is duplicative of the work of the reviewing IRB - Assessments of study feasibility are better performed by others (e.g., sponsors, scientific reviewers, investigators and/or their departments) | - Local knowledge is necessary to inform assessments of whether local resources are capable of manage the local application of a multisite protocol - Local sites can provide knowledge about the likelihood of meeting study accrual targets, given both characteristics of the site population as well as information about competing trials that may affect recruitment |

| Page Break |  |
| --- | --- |

1.3 Now that you’ve reviewed this information, please re-rate your response.
 
To what extent do you agree or disagree that **assessing study feasibility** (e.g., resources, staff, likelihood of meeting study accrual targets) should be a goal of local context review?

- Strongly disagree
- Disagree
- Unsure
- Agree
- Strongly agree

Please provide comments to explain your reasoning for your response (optional).

________________________________________________________________

________________________________________________________________

________________________________________________________________

________________________________________________________________

________________________________________________________________

| Page Break |  |
| --- | --- |

Question: To what extent do you agree or disagree that **promoting the quality of research** (for example, by tailoring research measures to be sensitive to cultural needs while maintaining scientific integrity) should be a goal of local context review?

Round 1 Responses:


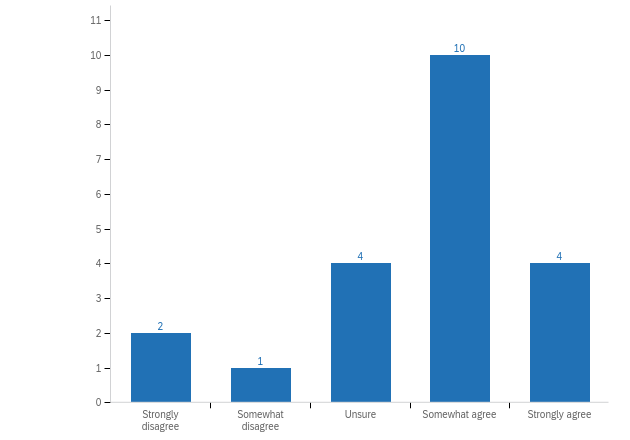


| **Qualitative Comments:** | |
| --- | --- |
| **Reasons for why it should NOT be a goal of LCR** | **Reasons for why it SHOULD be a goal of LCR** |
| - Responsibility for study quality falls on (and is better assessed by) the study team - This need not be considered at the local context, as it is already addressed by the reviewing IRB and sponsors and/or funding agencies | - A general goal of IRB/ethics review of studies is to facilitate quality research, so that research participants are not being burdened in the name of bad science, and because responsible use of limited research resources is an ethical imperative - Differences between study populations or sites might mean that one size does not fit all in terms of the specific ways research procedures are done or the specific measures are taken--failing to account for some of those differences might affect the quality of the science - Failing to account for the cultural needs and expectations of local populations can impede trial recruitment and retention, which in turn can undermine equity and representativeness |

| Page Break |  |
| --- | --- |

1.4 Now that you’ve reviewed this information, please re-rate your response.

To what extent do you agree or disagree that **promoting the quality of research** (for example, by tailoring research measures to be sensitive to cultural needs while maintaining scientific integrity) should be a goal of local context review?

- Strongly disagree
- Disagree
- Unsure
- Agree
- Strongly agree

Please provide comments to explain your reasoning for your response (optional).

________________________________________________________________

________________________________________________________________

________________________________________________________________

________________________________________________________________

________________________________________________________________

| Page Break |  |
| --- | --- |

1.5 Considering these four goals for local context review, please rate them in the order of most to least important.

______ Promoting the quality of research

______ Assessing study feasibility

______ Ensuring compliance with applicable laws and policies

______ Protecting the rights and welfare of local (relying site) participants

Please provide comments to explain your reasoning for your response.

________________________________________________________________

________________________________________________________________

________________________________________________________________

________________________________________________________________

________________________________________________________________

| Page Break |  |
| --- | --- |

**PART 2: Domains of Local Context**

In Round 1, you were asked to consider the types of information should be considered as part of local context review.
 
There was high agreement among expert participants that all four types of information explored should be considered as part of local context review.

In this round, we ask you to share your opinion as to how well you think current local context review processes consider each of these types of information.

After each rating you will be invited to share the reason for your rating.

| Page Break |  |
| --- | --- |

2.1 To what extent do you agree or disagree that current processes for local context review facilitate appropriate consideration for **population/participant-level characteristics (e.g., demographics, culture & religion, language & literacy, etc.)**?

- Strongly disagree
- Somewhat disagree
- Unsure
- Somewhat agree
- Strongly agree

Please provide comments to explain your reasoning for your response.

________________________________________________________________

________________________________________________________________

________________________________________________________________

________________________________________________________________

________________________________________________________________

| Page Break |  |
| --- | --- |

2.2 To what extent do you agree or disagree that current processes for local context review facilitate appropriate consideration for **investigator and research team characteristics (e.g., competency and training, credentialing, conflicts of interest, history of compliance issues or disciplinary actions, etc.)**?

- Strongly disagree
- Somewhat disagree
- Unsure
- Somewhat agree
- Strongly agree

Please provide comments to explain your reasoning for your response.

________________________________________________________________

________________________________________________________________

________________________________________________________________

________________________________________________________________

________________________________________________________________

| Page Break |  |
| --- | --- |

2.3 To what extent do you agree or disagree that current processes for local context review facilitate appropriate consideration for **institution-level characteristics (e.g., local standards of care, institutional policies, site-level resources and capabilities, etc.)**?

- Strongly disagree
- Somewhat disagree
- Unsure
- Somewhat agree
- Strongly agree

Please provide comments to explain your reasoning for your response.

________________________________________________________________

________________________________________________________________

________________________________________________________________

________________________________________________________________

________________________________________________________________

| Page Break |  |
| --- | --- |

2.4 To what extent do you agree or disagree that current processes for local context review facilitate appropriate consideration for **state and local laws (e.g., laws pertaining to age and decision-making, confidentiality, mandatory reporting, etc.)**?

- Strongly disagree
- Somewhat disagree
- Unsure
- Somewhat agree
- Strongly agree

Please provide comments to explain your reasoning for your response.

________________________________________________________________

________________________________________________________________

________________________________________________________________

________________________________________________________________

________________________________________________________________

| Page Break |  |
| --- | --- |

**PART 3: Permissible Exceptions from sIRB Requirements**
 
Some individuals have argued that local context considerations are so important for some types of studies that sIRB review would be inappropriate. Others have argued that sIRB review is inappropriate for some types of studies based on considerations of efficiency, adequacy of protections, or both.

In Round 1, we asked you to consider 13 proposed exceptions for the sIRB requirement.

In this round, we ask you to review 6 potential exceptions. For each, we have provided comments from both those who disagree and who agree that the study type described should receive an exception from the single IRB requirements (and therefore NOT required to use a single IRB).

After considering the responses and comments for your peers from Round 1, please re-rate the questions using the scale provided.

| Page Break |  |
| --- | --- |

Question: To what extent do you agree or disagree that research involving **unique ethnic or religious groups** should be granted an exception from the single IRB requirement?

Round 1 Responses:


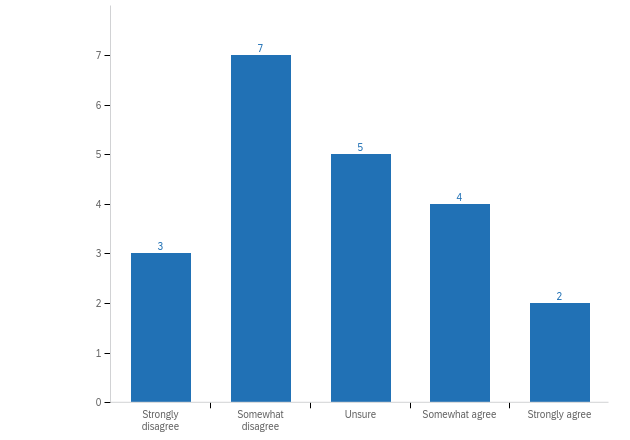


| **Qualitative Comments:** | |
| --- | --- |
| **Reasons provided by those who DISAGREE** | **Reasons provided by those who AGREE** |
| - The unique requirements of particular populations can be communicated to the reviewing IRB OR the reviewing IRB should be chosen based on their experience with the population - Research involving special populations might make sIRB review especially appropriate, as the reviewing IRB could be selected to ensure adequate expertise with research involving the relevant populations or groups | - “When some sites may involve unique ethnic or religious groups, there is an increased potential that centralized review (i.e., reviewing site) will be insensitive to or gloss over specific cultural considerations…” - “It would be very difficult for a [reviewing] IRB to have the IRB membership to review research that involves unique ethnic or religious groups…This is a challenge of [single] IRBs that an ad hoc member cannot meet” |

| Page Break |  |
| --- | --- |

3.1 Now that you’ve reviewed this information, please re-rate your response.
 
To what extent do you agree or disagree that research involving **unique ethnic or religious groups** should be granted an exception from the single IRB requirement?

- Strongly disagree
- Disagree
- Unsure
- Agree
- Strongly agree

Please provide comments to explain your reasoning for your response (optional).

________________________________________________________________

________________________________________________________________

________________________________________________________________

________________________________________________________________

________________________________________________________________

| Page Break |  |
| --- | --- |

Question: To what extent do you agree or disagree that research involving **a small number of sites (e.g., fewer than 5)** should be granted an exception from the single IRB requirement?

Round 1 Responses:


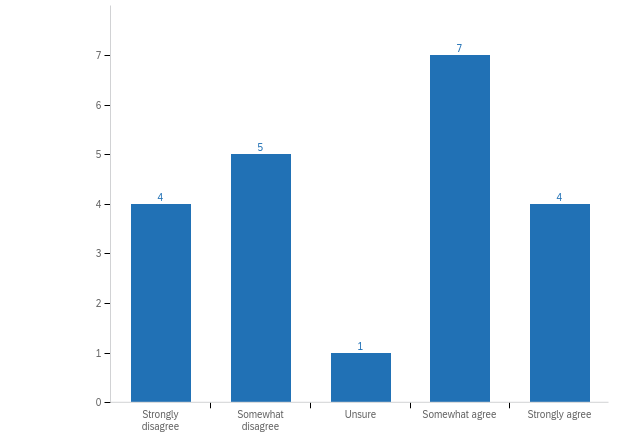


| **Qualitative Comments:** | |
| --- | --- |
| **Reasons provided by those who DISAGREE** | **Reasons provided by those who AGREE** |
| - The efficiency of a single IRB is not based on the number of sites; utility and efficiency may in fact be highest with a small number of sites, especially if sites are familiar with one another or frequently collaborate - Raises concern if a site starts with five but then chooses to add later (and may incentivize gaming the system to avoid single IRB review) | - The administrative work of reliance is likely to outweigh any benefits of centralizing review - The efficiencies gained by single IRB review diminish when there are a small number of sites, given the time and effort needed to establish reliance agreements |

| Page Break |  |
| --- | --- |

3.2 Now that you’ve reviewed this information, please re-rate your response.
 
To what extent do you agree or disagree that research involving **a small number of sites (e.g., fewer than 5)** should be granted an exception from the single IRB requirement?

- Strongly disagree
- Disagree
- Unsure
- Agree
- Strongly agree

Please provide comments to explain your reasoning for your response (optional).

________________________________________________________________

________________________________________________________________

________________________________________________________________

________________________________________________________________

________________________________________________________________

| Page Break |  |
| --- | --- |

Question:

To what extent do you agree or disagree that research involving **minimal risk studies** should be granted an exception from the single IRB requirement?

Round 1 Responses:


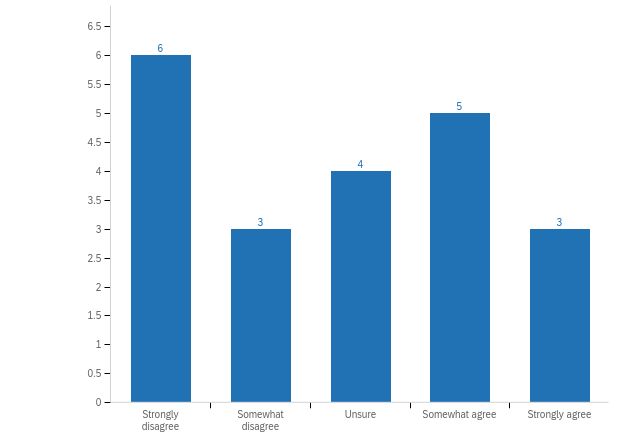


| **Qualitative Comments:** | |
| --- | --- |
| **Reasons provided by those who DISAGREE** | **Reasons provided by those who AGREE** |
| - The determination of minimal risk may vary across sites; single IRB review can standardize this determination - The level of risk should not be a predominant factor for meriting an exception - Minimal risk studies seem well-suited for centralized review, as there are fewer worries about the adequacy of protection | - Minimal risk studies can be reviewed faster individually by each site than the time needed to establish reliance agreements and related requirements for single IRB review - Single IRB start-up is inefficient; minimal risk studies have no continuing review, and any amendments can often be reviewed via expedited procedures, thereby undermining any efficiency gains from centralized review |

| Page Break |  |
| --- | --- |

3.3 Now that you’ve reviewed this information, please re-rate your response.
 
To what extent do you agree or disagree that research involving **minimal risk studies** should be granted an exception from the single IRB requirement?

- Strongly disagree
- Disagree
- Unsure
- Agree
- Strongly agree

Please provide comments to explain your reasoning for your response (optional).

________________________________________________________________

________________________________________________________________

________________________________________________________________

________________________________________________________________

________________________________________________________________

| Page Break |  |
| --- | --- |

Question: To what extent do you agree or disagree that research involving **research studies that are not clinical trials, including epidemiological or social science research**, should be granted an exception from the single IRB requirement?

Round 1 Responses:


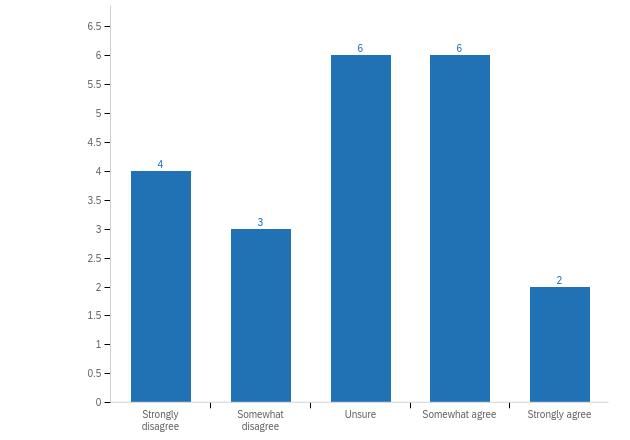


| **Qualitative Comments:** | |
| --- | --- |
| **Reasons provided by those who DISAGREE** | **Reasons provided by those who AGREE** |
| - Unless there are other extenuating circumstances, no evident reason why an exception would be beneficial - As long as there is local context review to ensure local requirements are met, single IRB review is acceptable | - These types of studies can often be reviewed quickly locally. The time taken to set up reliance agreements often exceeds the time needed for local individual review - The single IRB process is best suited for the review of clinical trials; reviewing epidemiological or social science research adds “complexity and considerable work -- for no real gain” |

| Page Break |  |
| --- | --- |

3.4 Now that you’ve reviewed this information, please re-rate your response.
 
To what extent do you agree or disagree that research involving **research studies that are not clinical trials, including epidemiological or social science research**, should be granted an exception from the single IRB requirement?

- Strongly disagree
- Disagree
- Unsure
- Agree
- Strongly agree

Please provide comments to explain your reasoning for your response (optional).

________________________________________________________________

________________________________________________________________

________________________________________________________________

________________________________________________________________

________________________________________________________________

| Page Break |  |
| --- | --- |

Question: To what extent do you agree or disagree that research involving **non-clinical studies in which the sites are not conducting the same research activities*** should be granted an exception from the single IRB requirement?

Round 1 Responses:

 
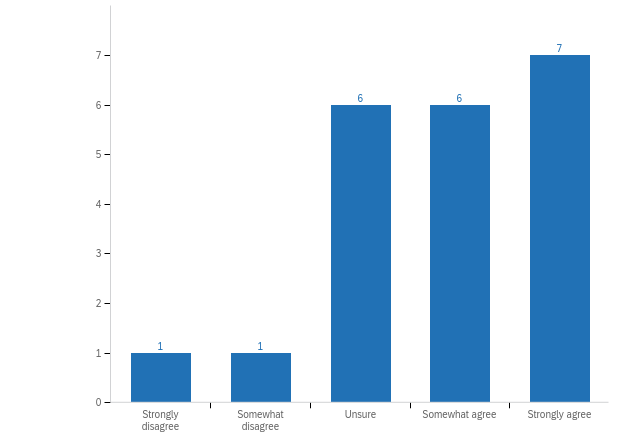


| **Qualitative Comments:** | |
| --- | --- |
| **Reasons provided by those who DISAGREE** | **Reasons provided by those who AGREE** |
| - There is no advantage to multiple IRBs if all the data/results are being used for the same research question - This may depend on the capacity of the reviewing IRB. If the reviewing IRB is conducting all research activities at their institution or is a commercial IRB, they likely could manage this study type. Otherwise, it could be difficult to manage and may merit an exception. | - The advantage of using a single IRB is directly related to similar procedure being done at multiple participating sites. When there are different activities in a multi-site study being done at different sites, it reduces the advantages with using a single IRB and introduces advantages that are associated with using local IRBs rather than a single IRB. - Requiring use of a single IRB for these types of studies is “akin to performing multiple reviews but in a less efficient way.” Single IRB is only useful when all sites are conducting identical tasks. |
| *****For example, consider a study involving both qualitative interviews and quantitative surveys. The team at Site A has social science expertise and will conduct qualitative interviews with participants at their site to inform the development of an online survey that will be administered by collaborators at Site B. The research team at Site B has expertise in survey design and analysis and will have access to a large study population at their institution. Therefore, the Site B team will administer the survey to the study population recruited from their institution and conduct the data analysis.  Researchers at Site A do not need, nor will have access, to the survey data collected at Site B. | |

| Page Break |  |
| --- | --- |

3.6 Now that you’ve reviewed this information, please re-rate your response.
 
To what extent do you agree or disagree that research involving **non-clinical studies in which the sites are not conducting the same research activities** should be granted an exception from the single IRB requirement?

- Strongly disagree
- Disagree
- Unsure
- Agree
- Strongly agree

Please provide comments to explain your reasoning for your response (optional).

________________________________________________________________

________________________________________________________________

________________________________________________________________

________________________________________________________________

________________________________________________________________

| Page Break |  |
| --- | --- |

Question: To what extent do you agree or disagree that research involving **studies operating under EFIC (exception from informed consent for emergency research)**should be granted an exception from the single IRB requirement?

Round 1 Responses:


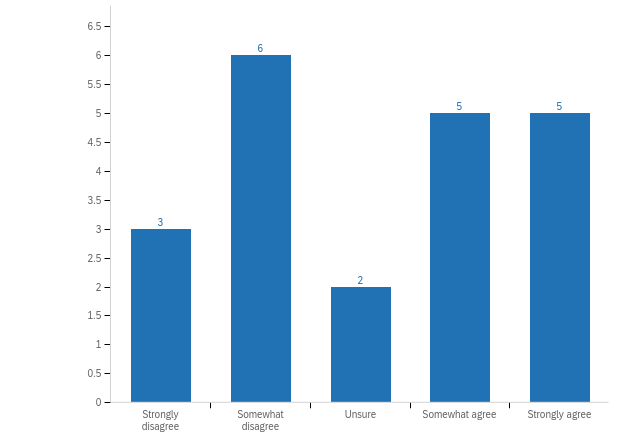


| **Qualitative Comments:** | |
| --- | --- |
| **Reasons provided by those who DISAGREE** | **Reasons provided by those who AGREE** |
| - “Equity and ability to compare local context from different sites as well as concentrated IRB experience…outweigh potential benefits of excluding from central review” - Recent examples have demonstrated sIRBs can appropriately review EFIC studies - The community outreach required by EFIC can be done locally and communicated to the sIRB | - The requirement for a community consultation plan for EFIC studies makes sIRB a “very awkward instrument” and/or “not efficient” for these studies - EFIC studies require community review at each site. The need for this local community input makes it more efficient for review to be done individually at each site. |

| Page Break |  |
| --- | --- |

3.7 Now that you’ve reviewed this information, please re-rate your response.

To what extent do you agree or disagree that research involving **studies operating under EFIC (exception from informed consent for emergency research)** should be granted an exception from the single IRB requirement?

- Strongly disagree
- Disagree
- Unsure
- Agree
- Strongly agree

Please provide comments to explain your reasoning for your response (optional).

________________________________________________________________

________________________________________________________________

________________________________________________________________

________________________________________________________________

________________________________________________________________

| Page Break |  |
| --- | --- |

If there are additional issues related to local context review that you think are important to consider, we invite you to share your thoughts below:

________________________________________________________________

________________________________________________________________

________________________________________________________________

________________________________________________________________

________________________________________________________________

End of Block: Default Question Block

Delphi Round 3

Start of Block: Default Question Block

INTRODUCTION

Thank you for completing the first two surveys in this Delphi study of local context review by single IRBs. Your ratings and comments are invaluable to this process.

In this third and final round, you will be asked to do two things:

1. REVIEW the draft Final Report.

2. SHARE YOUR FEEDBACK on the report’s findings, via the following short survey

| Page Break |  |
| --- | --- |

INSTRUCTIONS

Please complete this survey on a computer (as opposed to your phone) due to the length and complexity of the survey.

You are permitted to page back and forth in the survey using the arrows at the bottom.

We anticipate it will take you approximately one hour to review the report and complete this survey.

We recommend that you complete this survey in one sitting. However, if you need to leave the survey and return to it at another time, you may do so by leaving the survey and re-clicking on the link in your email when you are ready to return. All of the work that you have done up to that point will have been saved.

Your careful attention and commitment to a thoughtful response is greatly appreciated. As an expression of our appreciation for your time and input, you will be reimbursed $100 for completion of this survey.

If you have any questions, please reach out to Stephanie Morain at smorain1@jhu.edu.

| Page Break |  |
| --- | --- |

**PART 1:**

**Goals of Local Context**

In Rounds 1 and 2, you were asked to consider the goals of local context review. You were also asked to provide comments to explain your ratings.

In this round, we ask you to suggest improvements for how local context review might better promote two such goals.

| Page Break |  |
| --- | --- |

1. What improvements, if any, might you suggest for how local context review could better promote the goal of:

(a) protecting the rights and welfare of local (relying site) participants?

___________________________________________________________

___________________________________________________________

___________________________________________________________

___________________________________________________________

___________________________________________________________

(b) ensuring compliance with applicable laws and policies?

___________________________________________________________

___________________________________________________________

___________________________________________________________

___________________________________________________________

___________________________________________________________

| Page Break |  |
| --- | --- |

**PART 2: Domains of Local Context**

In Rounds 1 and 2, you were asked to consider (a) what types of information should be considered as part of current context review, and (b) how well local processes facilitate consideration of those information types.

In this round, we ask you to suggest improvements for how local context review might better facilitate adequate consideration of relevant information.

| Page Break |  |
| --- | --- |

2. What improvements, if any, might you suggest for how current processes for local context review might better facilitate adequate consideration of:

(a) population/participant-level characteristics?

___________________________________________________________

___________________________________________________________

___________________________________________________________

___________________________________________________________

___________________________________________________________

(b) investigator and research team characteristics?

___________________________________________________________

___________________________________________________________

___________________________________________________________

___________________________________________________________

___________________________________________________________

(c) institution-level characteristics?

___________________________________________________________

___________________________________________________________

___________________________________________________________

___________________________________________________________

___________________________________________________________

(d) state and local laws?

___________________________________________________________

___________________________________________________________

___________________________________________________________

___________________________________________________________

___________________________________________________________

| Page Break |  |
| --- | --- |

**PART 3: Permissible Exceptions from sIRB Requirements**

In Rounds 1 and 2, you were asked to consider proposed exceptions from the requirement to use a sIRB.

In this round, we ask you to select among three approaches for how exceptions, if any, should be managed.

You will then be asked to offer any additional reflections or recommendations related to the overall study.

| Page Break |  |
| --- | --- |

3. Which of the following comes closest to your view?

- Exceptions to the sIRB requirement should never be permitted.
- Exceptions to the sIRB requirement should be permitted for certain categories of studies (such as those categories explored in Part 3, e.g., those involving a small number of sites, those conducted under EFIC, etc.)
- Exceptions to the sIRB requirement may be appropriate for some studies, but should be made on a case-by-case basis.

| Page Break |  |
| --- | --- |

**Additional Questions:**

4. Now that you’ve reviewed the report, please offer any additional reflections or recommendations you have related to how to improve local context review for sIRBs.

___________________________________________________________

___________________________________________________________

___________________________________________________________

___________________________________________________________

___________________________________________________________

5. If you would like to be recognized by name in the “acknowledgements” section of any resulting publication or presentation, please type your name as you would like it listed:

___________________________________________________________

End of Block: Default Question Block
